# A state-based, proportional myoelectric control method: online validation and comparison with the clinical state-of-the-art

**DOI:** 10.1186/1743-0003-11-110

**Published:** 2014-07-10

**Authors:** Ning Jiang, Thomas Lorrain, Dario Farina

**Affiliations:** 1 Department of Neurorehabilitation Engineering, Berstein Focus Neurotechnology Göttingen, Bernstein Center for Computational Neuroscience, University Medical Center Göttingen, Georg-August University, Von-Siebold-Str. 6, Göttingen 37075, Germany; 2 RMS Signal & Innovation, BP 40054, Aix en Provence, Cedex 3 13792, France

**Keywords:** Prosthetic control, Electromyography, Signal processing, Proportional control, Pattern recognition

## Abstract

**Background:**

Current clinical myoelectric systems provide unnatural prosthesis control, with limited functionality. In this study, we propose a proportional state-based control method, which allows switching between functions in a more natural and intuitive way than the traditional co-contraction switch method.

**Methods:**

We validated the ability of the proposed system to provide precise control in both position and velocity modes. Two tests were performed with online visual feedback, involving target reaching and direct force control in grasping. The performance of the system was evaluated both on a subject with limb deficiency and in 9 intact-limbed subjects, controlling two degrees of freedom (DoF) of the hand and wrist.

**Results:**

The system allowed completion of the tasks involving 1-DoF with task completion rate >96% and of those involving 2-DoF with completion rate >91%. When compared with the clinical/industrial state-of-the-art approach and with a classic pattern recognition approach, the proposed method significantly improved the performance in the 2-DoF tasks. The completion rate in grasping force control was >97% on average.

**Conclusions:**

These results indicate that, using the proposed system, subjects were successfully able to operate two DoFs, and to achieve precise force control in grasping. Thus, the proposed state-based method could be a suitable alternative for commercial myoelectric devices, providing reliable and intuitive control of two DoFs.

## Background

There has been considerable effort in research and development of powered replacement prostheses for upper extremities. Myoelectric controlled prostheses have been investigated in research projects for many years [1]. In the past few decades, numerous studies have been published on myoelectric control for upper extremities (for a recent review, refer to [2]). Systems based on the state-of-the-art academic algorithms can classify multiple hand functions in laboratory settings, but have not yet been translated into commercial/clinical products, mainly due to their limited reliability. Commercial myoelectric controlled hand prostheses, which provide two degrees of freedom (DoF), rely on co-contractions to switch between the DoFs [3], which is not intuitive. To increase the acceptance rate of the users, a more intuitive control method of multifunction prostheses is needed [4].

The main focus in academic myoelectric control research has been pattern recognition. Pattern recognition systems are trained to recognize patterns in the surface electromyogram signals (EMG) and to select the corresponding function to execute. While they have been extensively tested in laboratory conditions, showing promising results, their impact in clinical applications remains limited outside the small group of patients who undergone targeted muscle reinnervation (TMR) surgery [5,6], which provided enough intuitive signal sites for these patients who otherwise had none. A few attempts were made in order to increase the reliability of pattern recognition systems, using approaches such as majority vote [7] or velocity ramp [8]. To some extent, these methods have improved the reliability of pattern recognition based algorithms, but at the cost of reduced prosthesis reaction time and anyway not at a level satisfactory for clinical large-scale applications.

In this study, we present a state-based algorithm for myoelectric control that aimed at improving the user performance with respect to the myoelectric control systems implemented in commercial devices, when controlling the same functions of these devices (two DoFs) [3]. Rather than using muscle co-contraction to switch between DoFs, as in classic commercial prostheses [3], the proposed approach implemented a more natural control for the switch between functions, and an adaptive proportional control of the activated functions. We validated the new method with online goal-directed tasks with real time feedback, both on able-bodied subjects and on a subject with limb deficiency. The purpose of the validation was to determine if the proposed algorithm allowed subjects to reach reliable and precise target position and force control.

## Methods

### Algorithm

**Figure 1:**
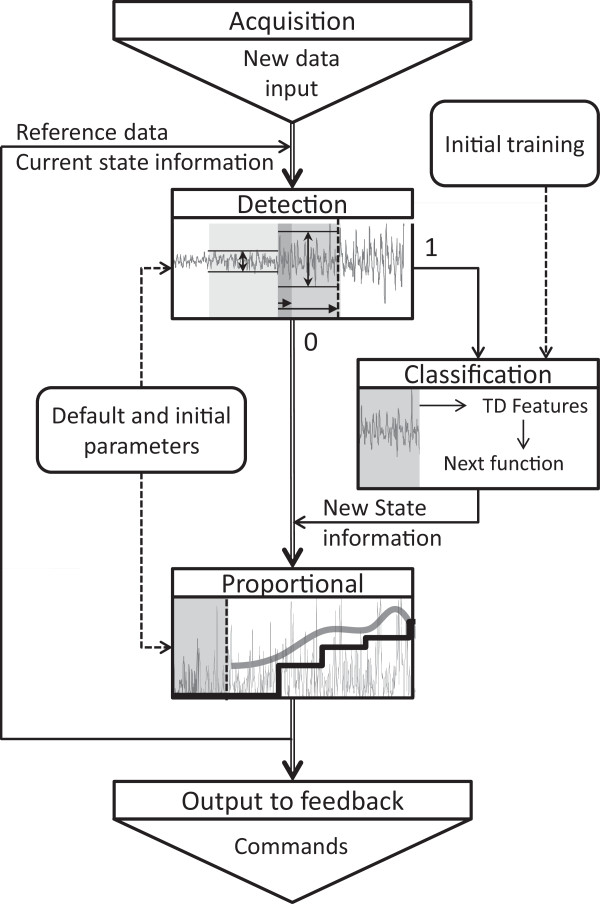
**Overall algorithm structure.** See text for detailed description.

The control algorithm used a state-based paradigm. In this framework, a “state” was assigned to each of the desired functions. The transitions among the different states were realized by detection and classification. For each state, proportional control was implemented. The schematic diagram of the processing framework is presented in Figure 1. The three main blocks of this framework are described in the following.

#### ***Detection step***

**Figure 2:**
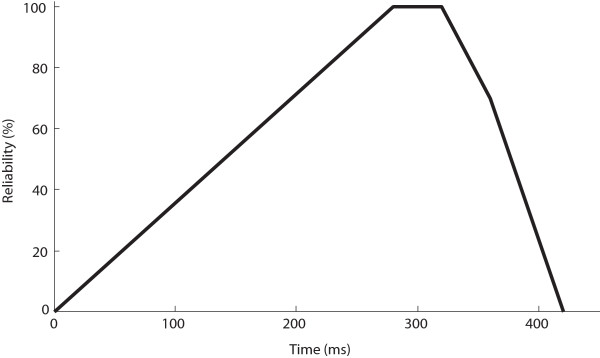
**The function *f***_***t***_ **(L) with its maximal value at 280 ms.**

The detection of transitions between motions was based on the average increase in variance across the surface EMG channels. The data were analyzed based on non-overlapping windows of 40 ms. Consecutive windows were used to form an analysis segment, whose length was variable. A measure of the change of variances (CV) in all channels between analysis segment and a reference buffer was computed as the following:

(1) $$\mathrm{CV}=\;\frac{1}{N}\sum_{i=1}^{N}\frac{\sigma_{AS_{i}}^{2}}{\sigma_{RB_{i}}^{2}}$$

where *N* is the number of channels, $\sigma_{AS_{i}}^{2}$ and $\sigma_{RB_{i}}^{2}$ are the variances of analysis segment and the reference buffer for channel *i*. The reference buffer stored the last 1500 ms data, or all available data from the last positive detection, whichever was shorter. This *CV* was subsequently compared to a variance ratio coefficient (VRC), to define a function of reliability of a motion transition (RMT):

(2) $$\mathrm{RMT}=\left\{\begin{array}{ll} f_{t}\left(L\right)\;\times \;\frac{\mathrm{CV}}{\mathrm{VRC}} & \mathrm{if}\;\mathrm{CV}<\mathrm{VRC}\\ f_{t}\left(L\right) & \mathrm{otherwise} \end{array}\right)$$

where *L* is the length of the analysis segment, and *f*_*t*_(*L*) is the function shown in Figure 2. The value of *VRC* was determined individually because the relative magnitudes of EMG signals generated by different subjects were substantially different. Once determined, it would be constant throughout the experiment for an individual subject. *RMT* was expressed in percentage. As the intended transition by the subject is usually short, the function *f*_*t*_(*L*) was introduced in order to reflect two scenarios: 1) the steady increase of the reliability with an impending motion transition when *L* is shorter than 300 ms; 2) the sharp decrease in reliability after 300 ms, in the event of a likely inadvertent action. The current *RMT* was then compared to a detection threshold (DT, see Results). When *RMT* > *DT* (positive detection) or *RMT* < 20% (no detection), the analysis segment would be reset. Otherwise, the next 40 ms of incoming data would be added to the analysis segment, and the procedure in (1) and (2) would be repeated. All parameters of the detection algorithm, with the exception of *VRC*, were determined across all subjects during preliminary tests.

#### ***Classification step***

Once a positive detection was triggered, the classification step would take place. In the current study, this step used a classic pattern recognition setting for myoelectric control [7]. The Hudgins time domain features (TD), *i.e.* mean absolute value, zero crossing, slope sign change and wave length [9], were used, and principal component analysis (PCA) was used to reduce the dimensionality of the feature space (components accounted 95% were kept). A linear discriminant analysis (LDA) was used as the classifier. The LDA was trained on the data from an initial training run (see Experimental protocol), where 12 s of training data (6 repetitions of 2 s contractions) were available for each motion. In the event of positive detection of motion transition, the most recent 240 ms of data prior to detection were used for classification of the next motion.

#### ***Proportional estimation***

The proportionality was calculated from the most recent data with variable window length (between 100 ms and 300 ms, adapted for subject’s comfort of use). Auto-regressive whitening was used for the surface EMG amplitude estimation, as it has been shown to improve the stability of amplitude estimates [10]. The 6-order autoregressive coefficients were obtained on the initial training data. Then motion-specific normalization of the mean absolute value (MAV) was applied to the whitened channels, providing instantaneous estimations of the intended proportional activation level. Based on the data obtained in the initial training run, a class-specific scaling factor was assigned to each channel for each class, and the MAV normalization was performed based on the current active class.

### Subjects

Nine able-bodied subjects (6 male and 3 female, 25–36 years old) participated in the experiments. Three of these 9 subjects had experience in myoelectric control systems whereas the other 6 subjects were naïve to EMG-related experiments. In addition to the intact-limb subjects, one subject with congenital limb deficiency (male, 24 years old) also participated in the same experiment. He has a left side malformation at the wrist and no wrist joint is present. The length of his left forearm is approximately 23 cm. He started using a regular myoelectric prosthesis 3 month prior to these experiments. The prosthesis uses the standard one-site-one-function control method, controlling hand open/close and wrist rotation with co-contraction as a switch mode. The experimental protocol was approved by the Research Ethics Committee of the University Medical Center Göttingen (#8/2/11). All subjects signed the informed consent before participation.

### Signal acquisition

**Figure 3:**
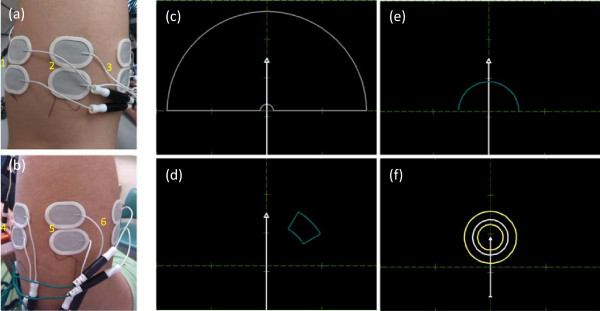
**The placement of the electrodes and the visual feedback interface. (a)** and **(b)** are an example of the electrode positions at the ventral and dorsal side of the forearm, respectively. The electrode numbers are marked in the photo. **(c)**-**(f)** are the visual feedback interface for the online performance tests. **(c)** the range of movement of the feedback arrow, with the arrow in neutral position (white arrow). Example of arrow in non-neutral position (dashed grey arrow). **(d)** Example of target during a target reaching attempt (right handed supination movement). **(e)** Grasping test initial setting, with the arrow in neutral position and object to grasp (dark blue line). **(f)** Grasping test force feedback once the object is reached. The white circle is the force feedback (diameter proportional to the force level, as estimated from the EMG), the yellow circles represent the upper and lower limit of the target force level. When the white circle moves outside large yellow circle which corresponds to the lower force limit, an ‘open’ function is activated.

Surface EMG were recorded using six pairs of electrodes (Ambu® Neuroline 720 01-K/12, Ambu A/S, Denmark) mounted on the dominant forearm. The electrode pairs were equally spaced around the forearm, one third distal from the elbow joint, similar to previous studies [11,12]. An example of the placement of the electrodes are shown in Figure 3a and b. The signals were recorded in monopolar derivation, with the reference electrode on the olecranon, amplified with a gain of 2000, filtered between 10 and 900 Hz, and sampled at 2048 Hz (EMG-USB2, OT Bioelettronica, Italy). All experiments were performed with the arm in neutral position (at the side of the body) and with the elbow close to full extension. The data were processed in real time, and online feedback was provided to the subject. The data processing period was 100 ms.

### Experimental protocol

Four essential functions (2 DoFs) of hand and wrist (closing/opening of the hand and supination/pronation of the wrist) were included in the experimental protocol, as they are the most important and functional functions for transradial amputees [13]. For the tests on the subject with limb-deficiency, the closing/opening of the hand functions were replaced by wrist extension/flexion, as requested by the subject. The experiment protocol was divided in three parts: initial training (1 run), target reaching test (3 runs), and grasping test (1 run).

The initial training run was performed at the beginning of the experiment, and consisted of a series of very short recordings (2 s per function). Six repetitions of each function, starting from rest, were performed, for a total of 48 s of training data. This training strategy included both transient and static data because of the strong transient nature of the signals to be classified after detection of a state change [12]. During this run, no feedback regarding the EMG activities was provided to the subject. After the training run, the training data were used to train the classifier (see Section “Algorithm”).

Once the classifier was trained, the two validation tests with online feedback were performed. The purpose of these two tests was to analyze different aspects of the proposed framework. The ‘Target reaching test’ was performed in velocity mode, and the ‘Grasping test’ was performed in position mode. The subjects were given 5 to 10 min to familiarize with the interface and the visual feedback, during which the proportional range of control for each class was optimized for the comfort of the subjects.

#### ***Target reaching test***

In this test, the ability of the algorithm to offer precise control was evaluated. The subject was presented with an arrow on a PC screen, as shown in Figure 3c). The arrow could rotate from -90 to +90 degrees, with the neutral position corresponding to zero degree. Such rotation corresponded to the functions “pronation” and “supination” of the wrist. The length of the arrow would increase with the “open” function, and decrease with the “close” function (see Figure 3c). In this test, the proportional control was operated in velocity mode, as it is done in most commercial prosthesis. The surface EMG signals of the subject controlled the velocity of the arrow feedback, i.e., when there was no surface EMG activity, the angle of the arrow and its length would not change. For each attempt, the arrow started in neutral position (see Figure 3c), and the subject was instructed (upon the change of color of the target from dark blue to yellow) to steer the arrow and place it within a 20% range (for both DoFs) of the target position (Figure 3d). This test consisted of 3 runs. During the first and second run, the target was placed in a position that could be reached by activating only one DoF (open/close or supination/pronation). These targets are referred to as 1-DoF targets. The angle and the radial distance of the targets were uniformly distributed over the full range in their respective ranges. An example of these target positions is represented in Figure 3d, for which only supination/pronation is necessary for accomplishing the reaching task. In the third run the target was placed such that it was necessary for the subject to control the two DoFs during the course of the attempt. The position of the target was determined randomly (uniform distribution) within the full range of the motions involved. These targets are referred to as 2-DoF targets. For each run, 30 targets were presented to the subjects, and the subjects were given 10s to complete each attempt. An attempt was considered successful if the target could be reached, and the arrow was maintained in the target for at least 1 s (dwell time) within the 10s allowed time.

#### ***Grasping test***

In the grasping test, the subject controlled in position mode, and only open/close was involved in the grasping test. Although no commercial prosthesis operates in this mode, position mode is more intuitive for tasks such as grasping objects than velocity mode. Therefore, it is important to investigate the position mode controllability of the proposed algorithm in grasping test. In this test, the ability of the algorithm to allow precise force control during grasping was evaluated. In this experiment, the subject needed to first activate the “closing” function to shorten the length of the arrow. When there is no EMG activity, the arrow would return to its original length. Before each trial, an object was displayed and the subject was instructed to reduce the length of the arrow (activating ‘close’ function), so that the tip of the arrow would reach the displayed object, represented by a circular blue line (see Figure 3e). Once the object was reached and as long as the “closing” function remained active, a white feedback circle centered on the arrow tip would be presented as shown in Figure 3f. The diameter of this circle would be proportional to the actual activation level of the ‘close’ function. Two additional yellow circles would be displayed (shown in Figure 3f), representing the target force range. The range of the target force was ±15% of maximum force for able-bodied subjects, and ±25% for the subject with limb deficiency. This range of target forces was chosen to simulate the limited target force variability necessary for a good force control in realistic scenarios. The target force was randomized (uniform distribution) between 20% and 60% of the maximal force. This run consisted of 30 attempts, in which the subject was given 20s to complete each attempt. An attempt was considered successful if the target force could be reached (and maintained within the target range for at least 1 s) within the 20 s.

#### ***Comparison with the clinical state-of-the-art***

To demonstrate the advantage of the proposed approach with respect to current commercial systems, 9 able-bodied subjects participated in an additional experiment, in which the industrial state-of-the-art (SOA) control method was used to perform the target reaching test. The industrial SOA utilized the one-site-one-function approach with two-control sites. Two pairs of electrodes (Ambu® Neuroline 720 01-K/12, Ambu A/S, Denmark) were placed over the flexors and extensors of the wrist, respectively. The signal was recorded in differential mode, resulting in two surface EMG channels, as the electrodes used in most commercial prostheses [14]. Prosthetic experts selected the sites with the criterion of minimal cross-talk between the two channels. An activation threshold was set for each channel. When the threshold of the channels was exceeded, the corresponding function would be selected (e.g., supination or pronation). When the two thresholds were simultaneously exceeded, a mode switch would take place (e.g. from rotation mode to open/close mode). Individual thresholds of the two channels were chosen through the standard procedure in prosthetic fitting, such that occurrences of un-intended mode switches were minimal while intended activation commands could be easily articulated. The same algorithm was used in a recent online study [15].

#### ***Comparison with patter recognition***

To show the added advantage of the detection step over a pure pattern recognition method, the 9 able-bodied subjects also participated to an experiment session in which they used a pure pattern recognition-based myoelectric control to perform the same target reaching tests. The experimental setup was identical to that of the proposed method, including electrode placement, data acquisition, and online control paradigm. In addition to the four classes used in the main experiment, a rest class (no movement) was also included. The classic time domain (TD) features were extracted from the raw EMG, and a linear discriminant analysis (LDA) was used as the classifier. A 3-vote majority vote (MV) was also implemented [16]. The pure pattern recognition algorithm is the implementation as in [7], and it is shown to be the academic SOA pattern recognition algorithm [2].

Both of the above comparisons were performed on different days from the main experiment, due to the duration of the experiment.

#### ***Performance metrics***

For both ‘target reaching test’ in velocity control mode and ‘Grasping test’ in position control mode, the performance metrics included the task completion rate, and task completion time for successful attempts. In the target reaching test, task completion time was computed from the onset of EMG activity during the corresponding attempt. To quantify the added difficulty from 1-DoF tasks to 2-DoF tasks, the normalized increase in completion time (nICT) was calculated as the difference between the average completion time in the two cases divided by the average 1-DoF task completion time. This index was used for the three investigated algorithms: the proposed method, industrial SOA, and pattern recognition.In the grasping test, the task completion time was computed from the instant of switch to direct force control. Two types of error could occur during this test. A misclassification occurs when the classifier output is either of the two rotation classes. In a realistic scenario, this type of error is likely less detrimental than grasp failure. Grasp failure happens when the classifier output is ‘open’, the opposite of the correct class. In a realistic scenario, this error would immediately lead to a ‘grasp failure’. As such, two additional metrics were used for the grasping test: misclassification rate during grasping and grasp failure rate. There is another possible error in the grasping test, which would occur when the exerted force exceed the upper limit of the target force (the inner circle in Figure 3f). However, this rarely happened, and was not reported.

### Statistical analysis

For target reaching test, paired *t*-test was performed on task completion rate and task completion time, respectively, to assess if the two types of targets (1-DoF and 2-DoF) has a significant effect on the performance. For both 1-DoF and 2-DoF targets, separate one-way ANOVA with general linear model (GLM) was performed to investigate if user experiences (experienced user and naïve user) has any effect on the online control performance. To investigate the differences in the added difficulty from 1-DoF tasks to 2-DoF tasks, a repeated measure ANOVA was performed on the nICT index for the proposed method, the industrial SOA method, and the pure pattern recognition method.

For grasping test, one-way ANOVA with general linear model (GLM) was performed to investigate if user experience (experienced user and naïve user) has any effect on the online control performance.

## Results

**Figure 4:**
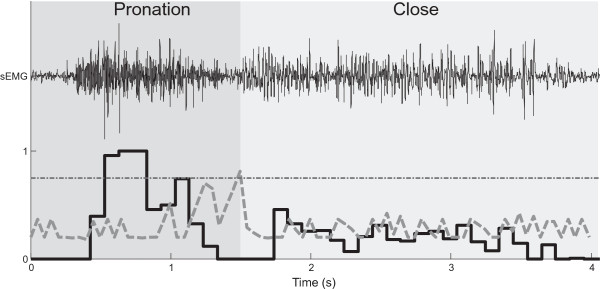
**Example of one channel of the surface EMG signals (top) along with the corresponding active functions (in colored shading) and proportional estimation (bottom black line) from one subject during a target reaching task.** The overlaying grey line is the RMT function as defined in Eqn. **(2)**, and the horizontal dashed line indicates the *DT* value.

A representative example of surface EMG (one channel), the active functions and proportional estimation during a target reaching tasks is illustrated in Figure 4. Note that at the system output, the transition between the two active classes was short (<300 ms).

### Target reaching test

**Figure 5:**
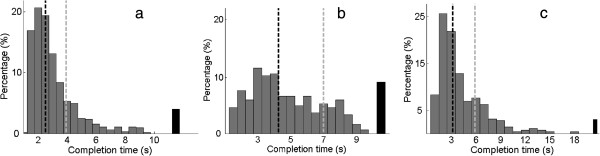
**Completion time histogram across all subjects for the targets in the target reaching tests. (a)** 1-Dof; **(b)** 2-Dofs; **(c)** the grasping test. The median and 80 percentile of the completion time are indicated by the black and grey dashed vertical lines, respectively. The black bar at the right end of each plot indicates the corresponding percentage task failure in each case.

In the target reaching test, for targets requiring only the activation of 1-DoF, the average completion rate across all subjects and across all classes was 96.2% ± 2% (mean ± std), with an average completion time of 3.0 ± 1.6 s. Across all individual classes, the lowest average completion rate was 94%, as shown in Table 1. The experienced subjects are indicated by (e), and the subject with limb deficiency by (a). The completion rate was greater on average for the experienced subjects (98.2 ± 2%) than for the naïve subjects (95.5 ± 2%). However, one-way ANOVA found the difference was not statistically significant (*p* = 0.14), likely due to the small number of subjects. Neither was the completion time statistically different (*p* = 0.43) between experienced subjects and naïve subjects (3.1 s ± 1.6 s and 3.0 s ± 1.6 s, respectively). The completion rate for the subject with limb deficiency was 94%, with an average completion time of 2.7 s ± 1.6 s. The histogram of the task completion time for 1-DoF target tasks is presented in Figure 5(a). The median and the 80 percentile of the task completion rate was 2.6 s and 4 s, respectively.

**Table 1:** Completion rate for all subjects

**Completion rate (%)**	**Open**	**Close**	**Supination**	**Pronation**	**1-Dof ave.**	**2-DoF ave.**
Subject 1 (e)	100	100	100	100	100	95
Subject 2	71	100	100	100	94	90
Subject 3	100	89	95	88	94	83
Subject 4	88	100	92	100	95	91
Subject 5	100	87	85	100	93	86
Subject 6 (e)	100	96	100	100	99	97
Subject 7	100	100	94	100	99	1
Subject 8	92	100	100	100	98	90
Subject 9 (e)	94	94	95	100	96	93
Subject 10 (a)	95	100	94	92	94	90
Average	94 ± 9	97 ± 5	95 ± 5	98 ± 4	96 ± 2	91 ± 5

Experienced able-bodied subjects are annotated by (e), the subject with limb deficiency by (a). The first four columns are the completion rate for the individual classes during 1-DoF tasks, the right most columns are the completion rate (average over all classes) for 2-DoF tasks.

For targets requiring the activations of both DoFs, the average completion rate was 91.5% ± 5%, lower than that of the 1-DoF tasks. Paired *t*-test found the difference significant (*p* < 0.01). Across all subject, the average completion time for these tasks was 4.8 s ± 2.3 s, higher than that of the 1-DoF tasks. Pair *t*-test found the difference significant (*p* < 0.01). The nICT index for the target reaching test was 0.59 ± 0.16, and was positive for all subjects. This indicates that the 2-DoF tasks were much more difficult than the 1-DoF tasks. Higher completion rate on average was again observed for experienced subjects (95.0% ± 2%) compared to naïve subjects (90.0% ± 6%), however the difference was not significant (*p* = 0.19). The completion time for 2-DoFs tasks was 4.5 s ± 1.9 s and 5.1 s ± 2.4 s for experienced and naïve subjects, respectively, however the difference was found to be not significant (*p* = 0.17). For the subject with limb deficiency, the completion rate was 90%, with a completion time of 4.2 s ± 2.0 s. The completion time histogram (see Figure 5(b)) indicated that the median and 80 percentile of the task completion rate were 4.3 s, and 7 s, respectively.

**Figure 6:**
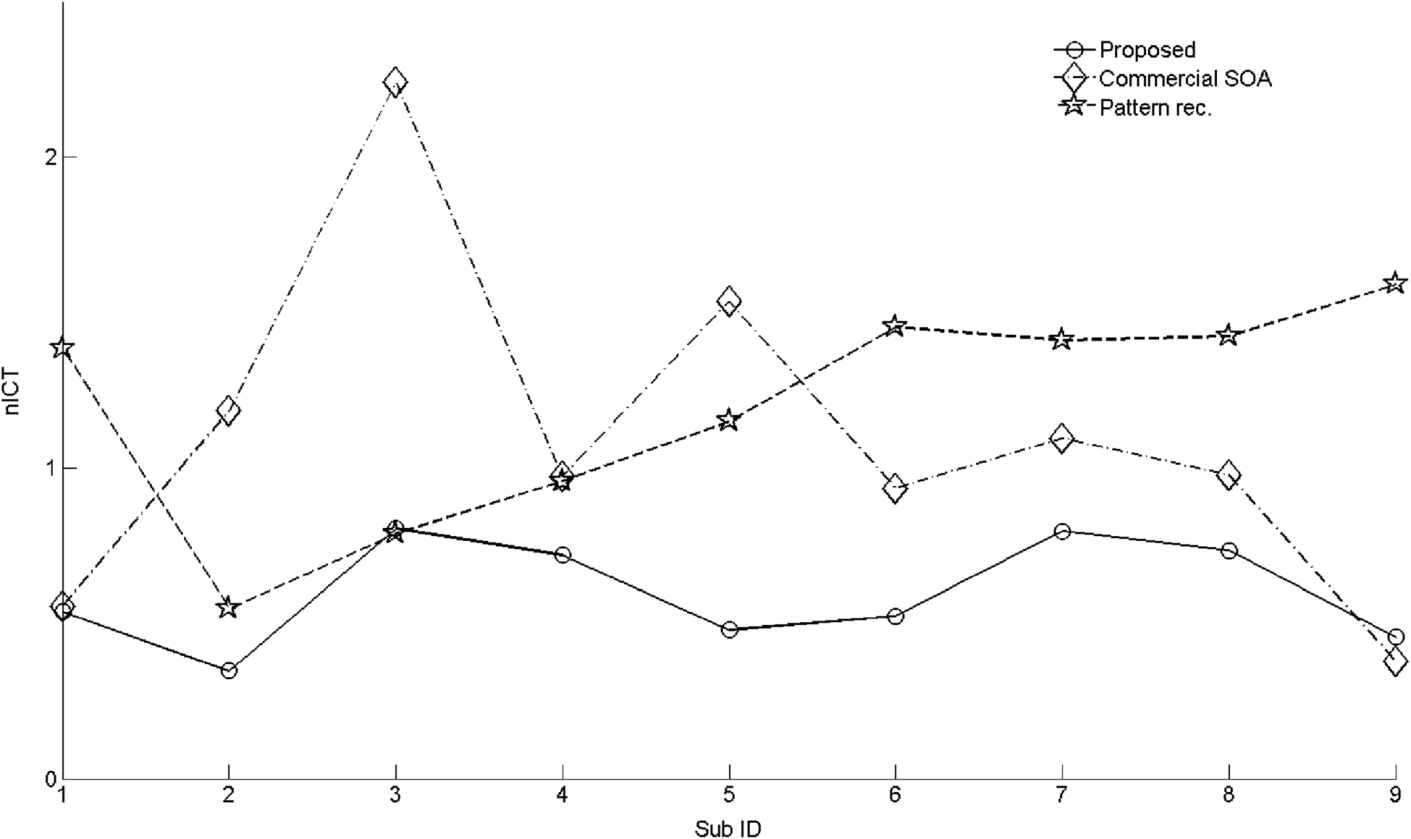
**The nICT index for the proposed method, the commercial SOA and the pure patter recognition method.** In all but one subject, the commercial SOA method resulted in a higher nICT than the proposed method. Similarly, the nICTs for only one subject was close for the proposed method and pure pattern recognition method. Repeated ANOVA revealed significantly different nICT for the three methods (*p* < 0.01).

In the industrial SOA experiment, the average task completion rate was 99.6% and 92.4% for 1-DoF task and 2-Dof task, respectively. The completion time was 2.69 s ± 0.58 s and 5.77 s ± 0.78 s, respectively. The nICT of the SOA approach was 1.22 ± 0.54. For the pure pattern recognition method, the 1-DoF task and 2-Dof task average completion rates were 98.9% and 95%, respectively. The completion time was 1.74 ± 0.66 s and 3.62 ± 0.90 s. Note that the average task completion rate and completion time for these two methods was either similar (industrial SOA) or even better than the proposed method (pattern recognition), as these two sessions were performed on a different day from the main experiment for each subject. This difference may due to various factors, including electrode placement, subject learning effect etc. The true advantage of the proposed method over the other two methods is demonstrated in the analysis of the nICT values, which reflects the added difficulty from 1-DoF tasks to 2-DoF tasks within the same experimental session (Figure 6). A repeated measure ANOVA showed that the method used had a statistically significant influence on nICT values. (*p* < 0.01). Tukey-Kramer post-hoc comparison revealed that the proposed method resulted in significantly smaller nICT values than both the industrial SOA and the pure pattern recognition methods. The nICT values of the latter two methods were not statistically different. This result demonstrated that, although 2-DoF tasks were more difficult for all three methods, the proposed approach made it significantly easier for the subject to perform 2-DoF tasks than the SOA approach and the pure pattern recognition method.

### Grasping test

In the grasping test, the average completion rate was 97% ± 5%, with an average completion time of 4.1 ± 2.8 s. Experienced subjects achieved on average higher completion rate (99% ± 2%) than naïve subjects (97% ± 5%), however the difference was not significant (*p* = 0.62). The completion time of experience subjects and naïve subjects was 3.2 s ± 2.2 s and 4.2 s ± 2.8 s, respectively, and the difference was significant (*p* = 0.006). The completion rate for the subject with limb deficiency was 89%, with a completion time of 5.6 s ± 2.4 s. The completion time histogram (Figure 5(c)) indicated that the median and 80 percentile of the task completion time were 3.5 s and 6 s, respectively. Table 2 reports the percentage of misclassification (classes other than close were activated) and grasping failure (open class was activated) per trial for each subject in the grasping test. The average misclassification per trial was 5% while grasp failure occurred in only 1% of all trials. The averages of misclassification and grasp failure went as low as 1% and 0%, respectively, for experienced subjects, while they were 6% and 2% for naïve subjects. The misclassification and grasp failure for the subject with limb deficiency were respectively 14% and 0%.

**Table 2:** Misclassification and grasp failure percentage per trial for each subject

**Subject**	**Misclassification (%/trial)**	**Grasp failure (%/trial)**
1 (e)	0%	0%
2	3%	3%
3	17%	6%
4	0%	0%
5	17%	0%
6 (e)	0%	0%
7	0%	0%
8	0%	0%
9 (e)	3%	0%
10 (a)	14%	0%
Average naïve (excl. a)	6% ± 8%	2% ± 2%
Average experienced	1% ± 2%	0%
Average overall (excl. a)	5% ± 7%	1% ± 2%

Experienced subjects are annotated with (e), and the subject with limb deficiency with (a).

## Discussion

We presented a novel state-based myoelectric control approach and the online control results from 9 intact-limbed subjects and one subject with upper limb deficiency using the proposed system. In the goal-directed online tests, the users would activate proportionally (sequentially) the two most important DoFs for trans-radial amputees: hand open/close and pronation/supination. The aim was to replicate the functionalities of commercial prostheses while providing a more natural switch between the two functions, without the need for an unintuitive and error-prone switching command such as co-contraction. Two online tests, a target reaching test, and a grasping test, were performed by the subjects. The target reaching test was chosen as it corresponds to one of the most representative tasks during the daily use of prostheses, i.e., reaching a precise target. In the current study, the subjects were free to operate the system as they preferred during the process of reaching, as opposite to tests based on sequences of predefined motions where the subject has very precise instructions at any point in time [17]. The completion rates for 1-DoF and 2-DoF target reaching tests were on average, respectively, 96.2% and 91.5%, demonstrating that precise positioning control can be achieved within a short time (average: 3.0 s and 4.8 s). Further, the proposed approach was shown to significantly reduce the difficulty of 2-DoF tasks, when compared with the industrial SOA approach and the pure pattern recognition method. The grasping test was chosen to evaluate the potential of the algorithm in force control. Grasping is the most relevant function for proportional force control, as the manipulation of different objects requires maintaining different force levels. The completion rate in the grasping test was above 97% on average, showing that the subjects were able to control force accurately using the proposed system. The time required to maintain the instructed force level was on average <4 s. Misclassification and, especially, grasping failure occurred rarely (Table 2). This indicates that the system has good reliability in grasping. For both tasks, the performance of the subject with limb deficiency was similar to the average performance of the able-bodied subjects.

The subject with limb deficiency, along with subjects #3 and #5, expressed tiredness towards the end of the experiment. This most likely accounted for their greater misclassification and/or grasp failure occurrences. It is important to note that comparing with other studies on the topic, the training data used in this study were very short (<1 min recordings for 4 active classes and resting class). This suggests that it is possible to recalibrate the proposed algorithm daily (or even more often when necessary). Alternatively, it is expected that adaptive extensions to the proposed algorithm could lead to an even better performance. For example, the parameter VRC could be adaptively varied to account for slow changes in the signal characteristics, which may occur because of factors such as fatigue.

The overall performance in all tests was better for the 3 experienced subjects compared to the 6 naïve subjects, although the differences were not statistically significant, likely due to the small subject sample. Nevertheless, it suggests that the results presented could be improved by subject training. The system indeed involves classification that requires the contractions to be as repeatable as possible to limit the variability between training and testing. Experienced myoelectric control subjects are “trained” to produce repeatable contractions to obtain the best results. Along with experience, the online feedback, as provided in this study, most likely facilitated the adaptation of the subject to the myoelectric system, and thus it is of primary importance in myoelectric control research [18]. For clinical applications, the improved performance with experience confirms that rehabilitation and training of the patients are key factors toward the successful use of myoelectric control for prosthesis control [19]. In this study, the results showed that, with experienced subjects, the proposed algorithm allowed very high performance in both reaching tasks (completion rate 1-DoF: >98%, 2-DoF: >95% on average) and grasping task (>98% on average), did not have any grasping failure, and resulted in nearly no misclassification during grasping. In addition, despite no experience in myoelectric control, the performance of naïve subjects was reasonably good. These results suggest that the proposed system offers intuitive control. The subject with limb deficiency showed comparable performance as the naïve able-bodied subjects. The lower completion rate in the grasping test is to be related with the specific anatomy of the subject, which results in very limited surface EMG activity during attempts to operate hand function or wrist flexion/extension. This accounts for the larger target range used in the grasping test with the subject with limb deficiency.

In the current state-of-the-art methods in pattern recognition based myoelectric control algorithms, most of the classification errors occur at the transition between classes, during which the misclassification of resting state into active motions has the most detrimental effect on the usability of the algorithms [12,20]. Various approaches have been proposed to address this problem. These include simple methods, such as majority vote [7], and more complex approaches, such as velocity ramp [8] and confident-based rejection [21,22]. The general approach of these methods is to utilize the history or prior information of the system, at the output of the classifier. The current approach exploited the prior information before the classification stage. The advantage of this approach is that the structure of the classifier is reduced because the detection stage effectively removes the necessity of a ‘resting class’, which was shown to be associated with the majority of classification errors [20]. Further, classification is only necessary when a state transition is detected, making the entire system more efficient. The direct comparison of the proposed method against the pure pattern recognition method (with MV post-processing) showed a significantly smaller nICT using the proposed method, supporting the advantage of the proposed method over a pure pattern recognition method.

The goal of myoelectric systems is to extract natural neural control information from EMG and to provide intuitive and reliable control of multiple functions to the prosthetic users. To this end, in this study we presented a real time state-based proportional control system. This system was shown to be reliable and to allow the subjects precise control of the feedback position and grasping force. With respect to commercial systems, the proposed algorithms provided a more natural and intuitive control, where the user can switch among available functions as it would be done naturally. This is in contrast with the conventional co-contraction based switching method, available in commercial systems, where the user needs to constantly monitor the active function, and perform additional strong contractions to switch between the available functions. Indeed, the subject with limb deficiency commented on the easiness of switching between wrist and hand functions of the proposed approach, as compared to the co-contraction switching mode in his commercial prosthesis. It is likely that the proposed approach would be more advantageous over the co-contraction-based switching when more functions need to be articulated since switching or circling through more than two functions would be mentally much more demanding.

In conclusion, we proposed a state-based myoelectric control system that was shown to be reliable and effective on the investigated situations, and to provide intuitive control to the subjects with minimal training. This system could provide a suitable alternative for the control of commercial prostheses.

## Abbreviations

DoF: Degree of freedom; EMG: Electromyography; TMR: Targeted muscle reinnervation; CV: Change of variance; VRC: Variance ratio coefficient; RMT: Reliability of motion transition; DT: Detection threshold; TD: Time domain; PCA: Component analysis; LDA: Linear discriminant analysis; MVA: Mean absolute value; SOA: State of the art; nICT: Normalized increase in completion time; ANOVA: Analysis of variance; GLM: General linear model; MV: Majority vote.

## Competing interests

The authors declare that they have no competing interests.

## Authors’ contributions

NJ and TL have equal contribution to the paper. NJ participated to the design and realization of the experiment, the data analysis, and the preparation of the manuscript; TL participated in the design of the study, conception, design and implementation of the algorithm, also carried out the experiments, data analysis, and the drafting of the manuscript; DF participated to the design and coordination of the study and to the manuscript preparation. All authors read and approved the final manuscript.
